# Assessing the conservation of Miombo timber species through an integrated index of anthropogenic and climatic threats

**DOI:** 10.1002/ece3.7717

**Published:** 2021-06-22

**Authors:** Silvia Catarino, Maria M. Romeiras, José M. C. Pereira, Rui Figueira

**Affiliations:** ^1^ Linking Landscape, Environment, Agriculture and Food (LEAF) School of Agriculture University of Lisbon Lisbon Portugal; ^2^ Forest Research Centre (CEF) School of Agriculture University of Lisbon Lisbon Portugal; ^3^ Centre for Ecology, Evolution and Environmental Changes (cE3c) Faculty of Sciences University of Lisbon Lisbon Portugal; ^4^ Research Centre in Biodiversity and Genetic Resources (CIBIO/InBIO) School of Agriculture University of Lisbon Lisbon Portugal; ^5^ Research Centre in Biodiversity and Genetic Resources (CIBIO/InBIO) University of Porto, Campus Agrário de Vairão Vairão Portugal

**Keywords:** Angola, climate change, conservation planning, Leguminosae, protected areas, species distribution models

## Abstract

**Aim:**

Angolan Miombo woodlands, rich in timber species of the Leguminosae family, go through one of the highest rates of deforestation in sub‐Saharan Africa. This study presents, on the basis of updated information of the distribution of Leguminosae timber species native to Angola, an integrated index framing the main threats for trees, which aims to support new conservation measures.

**Location:**

Sub‐Saharan Africa, Republic of Angola.

**Methods:**

The current distribution areas of six Leguminosae timber species (i.e., *Afzelia quanzensis*, *Brachystegia spiciformis*, *Guibourtia coleosperma*, *Isoberlinia angolensis*, *Julbernardia paniculata,* and *Pterocarpus angolensis)* were predicted through ensemble modeling techniques. The level of threat to each species was analyzed, comparing the species potential distribution with a threat index map and with the protected areas. The threat index of anthropogenic and climatic factors encompasses the effects of population density, agriculture, proximity to roads, loss of tree cover, overexploitation, trends in wildfires, and predicted changes in temperature and precipitation.

**Results:**

Our results revealed that about 0.5% of Angola's area is classified as of “Very high” threat, 23.9% as “High” threat, and 66.5% as “Moderate” threat. Three of the studied species require special conservation efforts, namely *B. spiciformis* and *I. angolensis,* which have a large fraction of predicted distribution in areas of high threat, and *G. coleosperma* since it has a restricted distribution area and is one of the most valuable species in international markets. The priority areas for the conservation of Leguminosae timber species were found in Benguela and Huíla.

**Main conclusions:**

This study provides updated data that should be applied to inform policymakers, contributing to national conservation planning and protection of native flora in Angola. Moreover, it presents a methodological approach for the predictions of species distribution and for the creation of a threat index map that can be applied in other poorly surveyed tropical regions.

## Graphical abstract

This study presents an integrated index framing the main threats for timber trees native to Angola. The current distribution areas of six Leguminosae species were predicted through ensemble modeling techniques and the level of threat to each species was analyzed, comparing the species potential distribution with a threat index map and with the national network of protected areas. We provide updated data to support the national conservation planning of native flora in Angola, and we develop a methodological approach that can be applied in other poorly surveyed tropical regions.

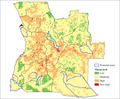

## 1 INTRODUCTION

The livelihood of many people in developing countries depends on natural resources for subsistence and income. Trees are one of the most valuable resources, as sources of food, medicine, wood, firewood, and charcoal (Djoudi et al., 2015).

In Angola, trees of the Leguminosae family, the most diverse plant family in the country, have been extensively exploited for different purposes (Catarino et al., 2019). They are among the most widespread species and are found mainly in Miombo woodlands, the dominant ecoregion in Angola and one of the major dry forest—savannas of the world (Burgess et al., 2004; Maquia et al., 2019). Angola is one of the richest African countries in terms of biodiversity and endemism (Goyder & Gonçalves, 2019); however, it also has one of the highest rates of deforestation in sub‐Saharan Africa (Hansen et al., 2003).

Timber species, and Angolan native flora in general, currently face many threats. Intensive wood harvest and illegal logging, wildfires, clearing lands for agriculture, urban expansion, and climate change were identified as the main threats (Catarino et al., 2019). At present, it is estimated that native forests occupy ca. 70 million hectares, but only 2.4 million hectares are considered productive forests capable of producing commercially valuable timber within a reasonable length of time (ACOM, 2019). Over the last years, the timber industry quickly increased, representing an important sector in the country's economy (Mendelsohn, 2019). However, the timber sector is still seen as a source of fast income and is dominated by “opportunistic companies” that do not manage the resource, but harvest it for quick profit (ACOM, 2019). Consequently, some native timber species are being heavily exploited by private companies without proper management and sustainable extraction plans (Chiteculo et al., 2018).

Angola is also one of the most vulnerable countries in sub‐Saharan Africa to climate change (Brooks et al., 2005). However, few data on the climate of Angola have been collected during the past four decades, due to the collapse of the network of weather stations maintained during the colonial times (Huntley, 2019). A recent study by Carvalho et al. (2017) provides the first analysis based on the climate change. Their study forecasts temperature changes of up to 4.9℃ by the end of the 21st century, with high impact in southeastern Angola. In contrast, they project a country‐wide mean decrease in precipitation of <2% for the same time frame, but the strongest change is also predicted for the southeast, with a decrease of up to 4% by 2,100. These changes will probably cause more frequent droughts, with high impacts on agriculture, water resources, and fire regimes (Carvalho et al., 2017).

Although wildfires are an important ecological factor in Miombo woodlands, substantial changes in natural fire regimes could be extremely destructive for native flora (Mendelsohn, 2019). Fire frequency has been increasing in recent years, and many anthropogenic fires have occurred in the late dry season, when the trees break dormancy and are more susceptible to damage (Catarino et al., 2020). Fires are also used in agriculture, in the traditional slash‐and‐burn technique, which consists of cutting trees and woody plants from an area and then burning biomass to fertilize the soil with the ashes and clean the area for cultivation and grazing (Schneibel et al., 2016; USAID, 2008).

The rapid economic and human population growth in Angola will increase the development of urban areas and the pressure on natural resources, with negative consequences for biodiversity and natural ecosystems (USAID, 2008). Considering that the country has a low adaptive capacity (Brooks et al., 2005), the study of ecosystems and species vulnerability to anthropogenic threats and climate change is critical to support the planning and implementation of conservation measures (Fremout et al., 2020).

The sustainable use of native species requires a vast knowledge of their life cycle and ecology, but the biodiversity of Angola remains poorly documented (Goyder & Gonçalves, 2019). Technical information on the exploited timber species is inexistent, and data on their distribution, ecology, and threats are very limited (Romeiras et al., 2014). In recent years, it has been possible to study the potential distribution of biodiversity in poorly surveyed countries such as Angola, through the application of species distribution models (SDMs) (Hernandez et al., 2008) These models are useful tools for management and conservation planning, including biodiversity assessment, reserve design, and habitat management (Sofaer et al., 2019). They are numerical techniques that extrapolate the potential species distribution in space and time, based on statistical models that find associations between environmental variables and species occurrences (Franklin, 2010). SDMs can perform well to characterize the natural distributions of species, particularly when relevant environmental variables are analyzed with an appropriate model (Elith & Leathwick, 2009).

In this study, we explored the level of threat to six Leguminosae timber species, based on their potential distribution in Angola estimated by an ensemble modeling approach. Legume species were selected as a case study because they are a good *proxy* for understanding conservation issues for useful plants as a whole. They belong to the largest plant family in Angola and have diversified into almost all terrestrial habitats, being a major component of the Miombo woodlands (Catarino et al., 2019; Olson et al., 2001). In addition, they improve soil fertility by fixing atmospheric nitrogen and are explored for a wide range of commercial applications, such as timber, charcoal, food, and medicinal products (Catarino et al., 2019).

The species potential distribution was compared with a threat index map composed of eight anthropogenic and climatic factors, and with the national network of protected areas. More specifically, we aimed to (a) provide new data on the distribution and conservation of the species studied; (b) analyze the spatial distribution of the main threats to native timber species of Angola, creating a threat map; (c) analyze the level of threat within the suitable area of occurrence of each species; and (d) investigate the adequacy and effectiveness of the national protected areas network to safeguard some of the most exploited timber species of the Angolan Miombo.

This study will provide updated information on the distribution and conservation of Angolan legume timber species and a geographic framing of the main threats for Angolan flora, which will be able to support new measures to ensure the preservation of the country's biodiversity. At the same time, we develop a methodological approach to inform conservation and management decisions that can be applied in other poorly surveyed countries.

## 2 MATERIALS AND METHODS

### 2.1 Study area

The study area corresponds to the Republic of Angola, located between 05°50′S and 18°02′S and 11°41′E and 24°05′E, excluding the northern enclave of Cabinda (Figure S1). The elevation, topography, river basins, proximity to the sea, and the cold current of Benguela have a strong influence on Angola's climate (Le Houérou, 2009). Average annual rainfall decreases from north to south and increases with altitude and distance from the ocean, ranging from ca. 50 mm in the Namib Desert to more than 1,500 mm in Lunda Norte (Huntley, 2019) The most representative ecoregion is the Angolan Miombo woodlands, with ca. 620,000 km^2^, occupying more than half of the country's area (Maquia et al., 2019).

Currently, the national protected areas’ network includes 14 terrestrial protected areas. Their geographic boundaries were obtained as GIS shapefiles from the World Database of Protected Areas (WDPA, 2020). The most recent protected areas are unavailable in WDPA and were vectorized based on the Angolan legislation (Diário da República de Angola, Law 38/11 on 29 December, 2011, p. 6340) using QGIS v.3.4.4 (QGIS & Development Team, 2020).

### 2.2 Species distribution modeling

#### 2.2.1 Leguminosae species data

**FIGURE 1:**
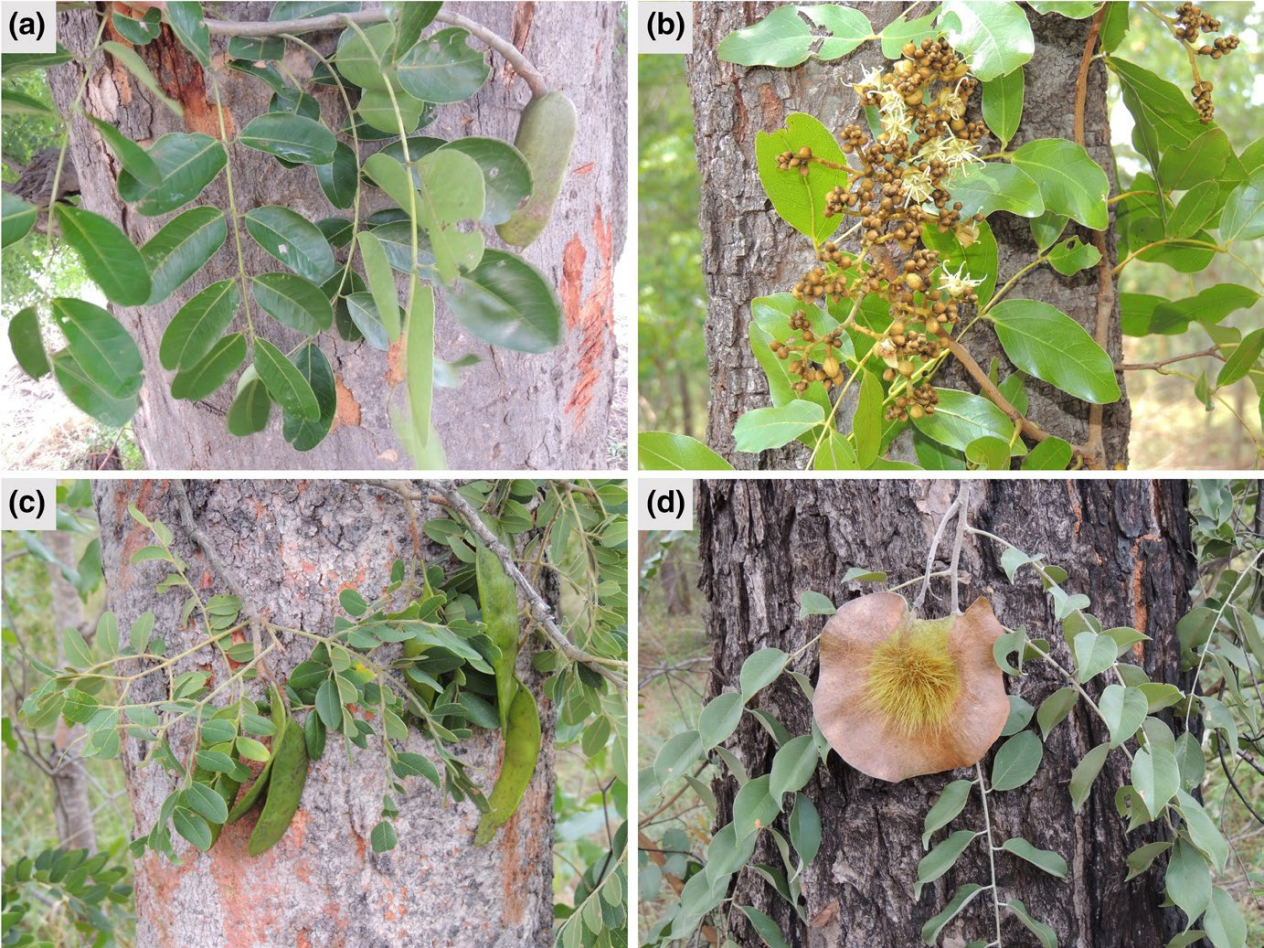
Leguminosae timber species highly exploited in Angola: (a) *Afzelia quanzensis* Welw.; (b) *Brachystegia spiciformis* Benth.; (c) *Guibourtia coleosperma* (Benth.) J. Léonard; and (d) *Pterocarpus angolensis* DC. Photographs by Luis Catarino

As a case study, we selected timber species from the Leguminosae family highly exploited in Angola and widespread in Miombo woodlands, namely *Afzelia quanzensis* Welw., *Brachystegia spiciformis* Benth., *Guibourtia coleosperma* (Benth.) J. Léonard, *Isoberlinia angolensis* (Welw. ex Benth.) Hoyle & Brenan, *Julbernardia paniculata* (Benth.) Troupin, and *Pterocarpus angolensis* DC (Figure 1, Table 1). Data on taxonomy, native distribution, main uses, and trade of each species were collected from recent studies focused on Angola flora (Catarino et al., 2019; Sanfilippo, 2013) and international databases [e.g., International Legume Database and Information Service (ILDIS, 2020), Plant Resources of Tropical Africa (PROTA, 2020), and Plants of the World Online (POWO, 2020)].

**TABLE 1:** The scientific name, local common name, wood trade name, global native distribution, main uses, and main type of trading of Leguminosae timber species native to Angola

Scientific name	Subfamily	Common name in Angola	English wood trade name	Native distribution	Main uses	Main trade	Ref. ^a^
*Afzelia quanzensis* Welw.	Detarioideae	Uvala, Mukungu	Pod mahogany	Southern DR Congo and Somalia, Angola, Botswana, Zimbabwe, Mozambique, and northern South Africa	Timber, charcoal, medicinal, food, forage, ornamental, honey plant	International and local markets	1, 3, 4
*Brachystegia spiciformis* Benth.	Detarioideae	Messasa, Mupanda	Zebrawood^b^	DR Congo, Kenya, Tanzania, and Angola to Zimbabwe, Mozambique, and northern South Africa	Timber, charcoal, medicinal, forage, fiber, tanning, honey plant	International and local markets	1, 2, 3, 4
*Guibourtia coleosperma* (Benth.) Leonard	Detarioideae	Musibi, Mussivi	African rosewood	Southern DR Congo, Zambia, Angola, Namibia, Botswana, and Zimbabwe	Timber, medicinal, food	International markets	3, 4
*Isoberlinia angolensis* (Welw. ex Benth.) Hoyle & Brenan	Detarioideae	Mutondo, Mone	Unknown	Southern DR Congo to Tanzania, Malawi, Zambia, and Angola	Timber, charcoal, medicinal	Local markets	1, 2, 3, 4
*Julbernardia paniculata* (Benth.) Troupin	Detarioideae	Olumue, Mumué	Unknown	Southern DR Congo, Angola, Tanzania, Zambia, Malawi, Zimbabwe, Mozambique	Timber, charcoal, medicinal, forage, honey plant	Local markets	1, 2, 4
*Pterocarpus angolensis* DC.	Papilionoideae	Girassonde	Kiaat	Southern tropical Africa, from Angola, DR Congo, Tanzania, South Africa, and Swaziland	Timber, medicinal, forage, fiber, dye, honey plant	International markets	1, 2, 3, 4

^a^ References: 1—Catarino et al. (2019); 2—Sanfilippo (2013); 3—PROTA (2020); 4—POWO (2020).

^b^ This commercial name refers to the wood of different African tree species.

All of the studied species are native to southern Africa, but *G. coleosperma* is of special concern since it has the most restricted distribution, mainly in Angola. From the bibliographic review (Catarino et al., 2019; PROTA, 2020; Sanfilippo, 2013), we observed that the selected species have several other uses besides timber production. For instance, *A. quanzensis* is used as forage and medicine for a wide range of health conditions; *G. coleosperma* has edible fruits and seeds, and its bark is a valuable treatment for skin diseases; and *P. angolensis* has several applications in medicine and the heartwood of its roots is used to produce a brownish‐red dye of great importance for tribal communities (Ndembu people in Angola). Charcoal production is another significant cause of overexploitation, especially for *A. quanzensis*, *B. spiciformis*, *I. angolensis*, and *J. paniculata*. *P. angolensis* and *G. coleosperma* are the most valuable species, mainly sold in international markets for construction, carpentry, and shipbuilding.

The occurrence records were gathered from GBIF—Global Biodiversity Information Facility (GBIF.org, 2020a, 2020b, 2020c, 2020d, 2020e, 2020f) and Santos (1982). Despite their limitations, the use of these data is justified by the lack of systematic field survey research (Elith & Leathwick, 2009). We downloaded the occurrence data recorded in all countries of sub‐Saharan Africa to improve the quality of the datasets and improve the contextual information for the models. Duplicated records (i.e., records with the same collector and the same collection number) were deleted, and the remaining records were analyzed in terms of quality and accuracy. For African countries except Angola, we only downloaded occurrence records with geographic coordinates, these records were projected on the map using Google Earth Pro 7.3.2.5491 (Serea, 2018), and it was checked whether the description of the collection site corresponded to the location projected on the map. When the points were more than 5 km away from the described location, they were excluded from the dataset. The occurrence data available for Angola are particularly scarce; thus, all the records collected in the country were downloaded and individually georeferenced, based on locality description and following the georeferencing protocol Guide to Best Practices for Georeferencing (Chapman & Wieczorek, 2006). Only records with less than 5 km of spatial uncertainty, determined as per the protocol, were included in the SDM.

As recommended by several studies (Araújo et al., 2019; Boria et al., 2014), we applied a 5‐km spatial filter to decrease sampling bias, reducing overfitting and increasing the models’ performance. This filtering method ensures that only one presence record is retained per 5 × 5 km grid cell, keeping the highest possible number of localities.

The final dataset, including 1,103 occurrence records [*A. quanzensis* (*n* = 172), *B. spiciformis* (*n* = 370), *G. coleosperma* (*n* = 112), *I. angolensis* (*n* = 79), *J. aniculata* (*n* = 143), and *P. angolensis* (*n* = 227) (Figure S2)], fulfilled the quality criteria proposed by Araújo et al. (2019) to model species distribution.

#### 2.2.2 Environmental variables

Based on previous studies and the expert knowledge of the species’ habitat and biology, we selected 25 ecological variables as possible predictors to fit the models, including 19 bioclimatic variables (Fick & Hijmans, 2017; Hijmans et al., 2005; WorldClim, 2020a), two solar radiation variables (Fick & Hijmans, 2017), three variables characterizing soil (Hengl et al., 2015), and elevation (Jarvis et al., 2008).

According to Burke, 2006) and Jinga and Ashley (2019), the bioclimatic variables derived from the monthly temperature and rainfall values have an important contribution in controlling the distribution of the studied species in Africa. Solar radiation is also known to play an important role in the distribution of most plant species and communities, mainly through photosynthetic activity. According to Austin and Van Niel (2011) and Piedallu and Gégout (2008), this factor is a necessary component of plant SDMs. Bioclimatic variables and mean solar radiation were downloaded from WorldClim 2.0 (WorldClim, 2020a) at a resolution of 2.5 arc minutes (ca. 5 km), as the mean of the years 1970–2000. This time frame is consistent with our dataset of occurrence records and is adequate for the studied species considering their long life span. Daily mean solar radiation data (kJ.m^−2^ day^‐1^) are available as monthly data, so we selected February (the month with the lowest values) and July (the month with the highest values) as potential predictors.

The distribution of the studied species is strongly associated with the Miombo woodlands, with highly leached, well‐drained, and usually acidic soils with a low organic matter content, and this ecoregion presents very specific edaphic conditions (Burgess et al., 2004). Thus, soil pH in H_2_O, and total nitrogen (N), and total phosphorus (P) contents of the soil fine earth were included as possible predictors for species distribution models. Recent studies (Buri et al., 2020; Dubuis et al., 2013) have shown that the geochemical variables of the soil significantly increased the predictive power of plant SDMs, and among the edaphic variables, pH and nitrogen content showed the highest effect. Soil data were downloaded from World Soil Information at a resolution of 250 m (ISRIC, 2020).

The elevation is also an important factor for the distribution of plant species in Angola, ranging between sea level and 2,620 m in the high mountains, and the omission of this factor would result in overpredictions of species suitable area (Oke & Thompson, 2015). Elevation data were downloaded from CGIAR‐CSI Consortium for Spatial Information (CGIAR‐CSI, 2020) at a resolution of 90 m. All environmental data layers were resampled to a spatial resolution of 5 km.

First, to avoid overweighting the analysis with bioclimatic variables, we performed an exploratory modeling exercise to investigate which ones were more suitable contributors to the SDM for each species. After 10 runs of each model technique, we used the “variables_importance” function to select the three variables related to temperature and the three variables related to precipitation that most contributed to the distribution model. Then, the variance inflation factor (VIF) (García et al., 2015; Mansfield & Helms, 1982) was used to assess and reduce multicollinearity between the remaining predictors, using R version 3.6.0. (R Development Core Team, 2020) and “usdm” package (Uncertainty Analysis for SDMs) version 1.1–18 (Naimi et al., 2014). The variable with the higher VIF value was rejected, and new scores were calculated until all the predictor variables scored lower than 10 (Neter et al., 1996). Table 2 presents the initial set of variables and the selected predictors.

**TABLE 2:** General description of environmental variables tested to build the species’ distribution models. The variables marked with “X” were selected to build the models of a specific species, after applying the variance inflation factor (VIF) to reduce collinearity

Environmental Variables	Code	Summary of variables^a^						Variables selected by species^b^					
		Mean	Min	Q1	Median	Q3	Max	AQ	BS	GC	IA	JP	PA
Annual mean temperature (°C)	BIO1	21.6	15.3	20.5	21.4	22.6	28.0		X				
Mean diurnal range [mean of monthly (max temp ‐ min temp)] (°C)	BIO2	14.2	5.9	13.2	14.6	16.0	18.6				X		X
Isothermality (BIO2/BIO7) (* 100) (°C)	BIO3	6.7	4.7	6.3	6.6	7.0	8.4	X	X			X	X
Temperature seasonality (standard deviation *100)	BIO4	186.2	28.5	121.1	182.3	254.9	355.6						
Max temperature of warmest month (°C)	BIO5	31.1	22.5	29.9	31.0	32.2	35.3				X	X	
Min temperature of coldest month (°C)	BIO6	9.5	3.8	6.3	8.4	12.3	20.4			X			
Temperature annual range (BIO5‐BIO6) (°C)	BIO7	21.6	12.5	17.6	21.9	25.8	28.9		X				
Mean temperature of wettest quarter (°C)	BIO8	22.7	16.3	21.3	22.4	23.9	28.6	X					
Mean temperature of driest quarter (°C)	BIO9	18.8	12.7	17.0	18.2	20.6	24.9						
Mean temperature of warmest quarter (°C)	BIO10	23.3	16.7	22.0	23.0	24.5	29.9						X
Mean temperature of coldest quarter (°C)	BIO11	18.8	12.5	16.9	18.1	20.5	24.9						
Annual precipitation (mm)	BIO12	1,020.2	21	751	1,092	1,296	1636						
Precipitation of wettest month (mm)	BIO13	202.3	14	184	211	228	300	X				X	
Precipitation of driest month (mm)	BIO14	0.5	0	0	0	0	17						
Precipitation seasonality (coefficient of variation) (mm)	BIO15	94.9	63	82	94	108	144	X	X			X	
Precipitation of wettest quarter (mm)	BIO16	523.3	17	451	551	618	770		X		X		X
Precipitation of driest quarter (mm)	BIO17	4.4	0	0	1	4	56						
Precipitation of warmest quarter (mm)	BIO18	311.2	26	219	288	390	624			X	X		X
Precipitation of coldest quarter (mm)	BIO19	10.6	0	2	4	13	668	X					X
Elevation above sea level (m)	Elevation	1,077.1	0	963	1,139	1,289	2,318			X		X	X
Soil pH in H_2_O	pH	5.8	4.6	5.4	5.6	6.0	9.1	X	X		X	X	X
Total nitrogen (*N*) content of the soil fine earth fraction in mg/kg (ppm)	Nutri_N	725.4	156	532	683	867	3,019	X	X	X	X	X	X
Total phosphorus (P) content of the soil fine earth fraction in mg/kg (ppm)	Nutri_P	318.9	14	224	282	363	2,613	X	X	X	X	X	X
Mean daily solar radiation in February (kJ.m^−2^ day^−1^)	Radia_02	17,151.4	13,786	15,461	16,773	18,770	21,298	X	X	X	X	X	X
Mean daily solar radiation in July (kJ.m^−2^ day^−1^)	Radia_07	16,583.3	9,851	16,108	17,312	17,683	18,453		X	X		X	X

^a^ Summary of variables: Min, minimum; Max, maximum; Q1, first quartile; Q3, third quartile.

^b^ Species: AQ, *Afzelia quanzensis*; BS, *Brachystegia spiciformis*; GC, *Guibourtia coleosperma*; IA, *Isoberlinia angolensis*; JP, J*ulbernardia paniculata*; and PA, *Pterocarpus angolensis*.

#### 2.2.3 Species distribution modeling

The potential distribution of each species was predicted through an ensemble modeling approach performed with the “biomod2” package (BIOdiversity MODelling‐Biomod2) version 3.3‐7.1 (Thuiller et al., 2020), implemented in R version 3.6.0 (R Development Core Team, 2020). Biomod2 is a computer platform for ensemble forecasting of species distributions, which allows maximizing the predictive accuracy of SDMs by combining different modeling methods (Araújo & New, 2007; Hao et al., 2019). We fitted the SDMs using an ensemble of five different modeling algorithms: two regression techniques, namely generalized linear models (GLMs) and multivariate adaptive regression splines (MARS); and three machine learning methods: generalized boosted models (GBMs), random forest (RF), and MaxEnt (detailed description of the model techniques in Elith et al., 2011; Franklin, 2010; Phillips et al., 2006). We selected these algorithms based on their superior performance during an exploratory modeling exercise.

All the selected modeling techniques require records of presence and absence, except MaxEnt, which is a presence background modeling tool. As our data are presence‐only, we generated three different sets of pseudo‐absences, where one‐third of the available background modeling cells were randomly sampled and used as pseudo‐absences, following the recommendations of Elith et al. (2006). The models were processed with the default settings for each modeling technique and the following options: equal weight of background absences and occurrences; 10,000 maximum interactions; 100 replicate runs for each set of “pseudo‐absences” and for each model technique; the occurrence data were randomly split into 70% training data and 30% test data to evaluate the predictive performance of the models; and 10 permutations to estimate variable importance. For each species were produced 1,500 individual models. Following the recommendations of Zurell et al. (2020), we present the ODMAP protocol with details of the modeling process in Data S1.

#### 2.2.4 Model evaluation

To evaluate model accuracies, we calculated the area under the curve (AUC) of the receiver operating characteristic (ROC) and the true skill statistic (TSS) using “models.eval.meth” function. The ROC curve is a threshold‐independent measure, which allows assessment of the predictive performance of the models by plotting sensitivity as a function of commission error (calculated as 1‐specificity), and AUC measures the area under this curve, relating the proportions of true positives (correctly classified cells) and false positives (incorrectly classified cells) over a continuous range of threshold levels (Peterson et al., 2008). AUC values range from 0.5 to 1, where values lower than 0.7 are considered low, between 0.7 and 0.9 are good, and higher than 0.9 are excellent (Manel et al., 2001). The TSS is a threshold‐dependent evaluation metric that assesses the ability of a model to correctly classify presences and absences (calculated as sensitivity +specificity ‐ 1), where sensitivity and specificity are calculated based on a defined threshold and are independent of species prevalence (Allouche et al., 2006). TSS ranges from −1 to +1, where zero or less indicate a performance no better than random, and values above 0.4 indicate a statistically reliable performance (Allouche et al., 2006).

The importance of variables was calculated using the “variables_importance” function that shuffles a single variable of the given dataset. It computes a correlation of the model predictions obtained with the initial dataset and with the shuffled dataset, and returns a result ranging from 0 to 1 (Thuiller et al., 2020). Higher values indicate stronger contribution of the variable to the model, and the value 0 indicates variable irrelevance.

#### 2.2.5 Ensemble models

The main advantage of ensemble methods is to reduce the production by the techniques with low predictive performance (Araújo & New, 2007). Only single models with AUC ≥0.7 were included in uncertainty of predictive single models by combining them and excluding the results ensemble model building. We chose the “mean” consensus method, as it is reported to provide significantly more robust predictions than the other consensus methods (Marmion et al., 2009). Finally, the predictive performances of ensemble models were evaluated with AUC and TSS metrics.

The continuous probability maps obtained were converted to binary maps of species presence and absence. Each pixel with a continuous value between 0 and 1 was reclassified into binary data by applying the 10th percentile threshold to define the suitable and unsuitable habitats. This threshold is widely used in species distribution modeling (Vale et al., 2014).

### 2.3 Map of threat index

The threat index map was created by combining different factors occurring in Angola. This approach was adapted from the terrestrial Human Footprint map proposed by Venter et al. (2016), following the methods originally developed by Sanderson et al. (2002). We included the same anthropogenic factors as the Human Footprint map, except those that have low relevance for the studied species or lack of data for Angola. Additionally, other factors related to deforestation and climate changes were included considering their high impact on timber species conservation. The final threat map includes eight factors for which we could assemble a national coverage, that is, population density, cropland areas, roads, overexploitation, loss of tree cover, increasing trends in burned area, and expected changes in temperature and precipitation.

Each threat layer was classified individually, the layers of population density and expected changes in temperature and precipitation were classified with a continuous scale, and the map cells can assume any value between 0 and 10, with a weighted value according to the intensity of the threat. The other layers were classified with discrete values, assuming the value of 0 when the threat is absent in the cell and the value of 10 when the threat is present (for more details, see Table 3).

**TABLE 3:** Summary of the eight individual factors used to build an index of threat

Threat factors	Years	Score	Details—reference threat value	Data source
Population density	2014	0–10 (continuous)	Cell value = 0.333 × log (population density +1)	INE (2014)
Cropland areas	2018	0 or 10	Cells with 20% or more covered by crops have the value 1.	ESA (2020)
Distance from roads	2018	0 or 10	Cells that buffer the primary and secondary roads up to a distance of 20 km have the value 1.	WFP (2018)
Overexploitation	2017	0 or 10	When the value of extraction licensed is higher than the adequate value of harvesting, the province has a value of 1.	ACOM (2020)
Tree cover loss	2000–2019	0 or 10	Cells with 20% or more of loss in the past 20 have a value of 1.	Global Forest Watch (2020)
Increasing trends in burned area	2001–2020	0 or 10	The significant areas of increasing trends of burned area and a buffer of 5 km have the value of 1.	Catarino et al. (2020)
Climatic changes in temperature	2041–2060	0–10 (continuous)	The value of each cell range increases linearly with the amplitude of changes, reaching the value of 1 when the temperature changes by 2℃ or more.	CMIP6 projections available on WorldClim (2020b)
Climatic changes in precipitation	2041–2060	0–10 (continuous)	The value of each cell range increases linearly with the amplitude of changes, reaching the value of 1 when the precipitation changes by 200 mm or more.	CMIP6 projections available on WorldClim (2020b)

Using the Quantum GIS 3.4 (QGIS Development Team, 2020), the selected factors were converted in raster files, resampled to 5x5 km spatial resolution by selecting the method of “Mean Value ‐ cell area weighted,” and rescaled with values from 0 to 10 in each cell of the grid layer. The resulting standardized factors were summed to create an exposure map. For any grid cell, the threat index could range from 0 (no threats) to 80 (maximum threats) and was categorized into four quantiles: “Low” (<20); “Moderate” (20–40); “High” (40–60); and “Very high” (>60).

Urban areas, obtained from the European Space Agency–Climate Change Initiative Land Cover (ESA‐CCI‐LC) project (ESA, 2020), were not included as a threat factor. These areas were excluded from species distribution maps and threat index map because they completely prevent the growth of natural vegetation.

Considering that the classification of threat layers was based on literature and expert opinion, we conducted a sensitivity analysis adapted from Fremout et al. (2020) to measure the effect of our methodological decisions on results. For this purpose, we created a “highest threat” map and a “lowest threat” map, with different values for each threat variable. The highest threat represents a scenario in which the threat threshold for the several factors is more restrict (lower) to the reference situation, and the lowest threat otherwise. These two maps were then compared with the reference map and provide a spatial indication of the variation of the impact that these factors have on the threat for timber species.

#### 2.3.1 Population density

Population density is used as a proxy for the pressure that human beings exert on the environment where they live. This factor was mapped based on the most recent census conducted in Angola (INE, 2014), which includes data at the municipality level. For the reference threat map, we assumed that the pressure induced by people increases logarithmically with increasing population density and saturates at a level of 1,000 people per km^2^, as suggested by Venter et al. (2016). The value of each cell was calculated on a logarithmic scale up to a maximum of 10, where pressure = 3.333 × log (population density + 1).

The saturation levels were adapted in the maps created for the sensitivity analysis. In the “highest threat” map, we assumed that the pressure saturates at a level of 800 people per km^2^ and the value of each cell was calculated as follows: pressure = 3.247 × log (population density + 1). In the “lowest threat” map, we assumed that the pressure saturates at a level of 1,200 people per km^2^ and was calculated as follows: pressure =3.444 × log (population density + 1).

#### 2.3.2 Cropland areas

We map cropland areas using recent land cover data (2018) obtained from the ESA‐CCI‐LC project (ESA, 2020), which produced annual land cover maps at 300‐m resolution, combining remote sensing products and ground observations. In the reference map, cells with ≥20% crop cover were classified with a value of 10. In the “highest threat” map, the value 10 was assigned to cells with ≥15% crop cover to increase the area of maximum threat and, in the “lowest threat” map, the value 10 was assigned only to cells with ≥25% crop cover to reduce the area of maximum threat.

#### 2.3.3 Distance from roads

Roads are important drivers of habitat conversion and fragmentation, reducing the extent of suitable habitats, increasing fire frequency, and providing easy access for humans. According to Oliveira et al. (2007), 75% of the total forest loss was detected at 20 km or less from the road. We obtained the map of the main roads from OpenStreetMap roads for the World Food Program (WFP, 2018). On the reference map, the grid cells that buffer the primary and secondary roads up to a distance of 20 km have the value 10; on the “highest threat” map, the buffer has 25 km; and on the “lowest threat” map, the buffer has 15 km.

#### 2.3.4 Overexploitation

The overexploitation layer was obtained by comparing the mean volume of extraction licensed in each province between 2015 and 2017, and the adequate value of harvesting estimated by the Angolan Institute of Forest Development, taking into account the species and their life span in each province (ACOM, 2020). When the value of the licensed extraction was higher than the adequate value of harvesting, the province was assigned a value of 10 in the reference threat index map. On the “highest threat” and the “lowest threat” maps, the province received a value of 10 when the licensed extraction was higher than the adequate value of harvesting minus 10% and plus 10%, respectively.

#### 2.3.5 Tree cover loss over the last 20 years

The identification of areas with a recent loss of forest cover allows to predict the future trend of forest cover and can give an overview of how deforestation will occur (Saha et al., 2020). The loss of tree cover was gathered from Global Forest Watch (2020), which includes the forest loss for the years 2000 to 2019. Cells with ≥20% of loss reported in their area over the past 20 years were assigned the value of 10. On the “highest threat” map and “lowest threat” map, cells with a ≥15% and ≥25% loss (respectively) were assigned the value of 10.

The inclusion of this factor may represent some redundancy with other layers considered in the index, namely cropland areas, and overexploitation.

#### 2.3.6 Increasing trends of burned area

The increasing trends of burned area are strongly related to changes in fire regimes. This factor is based on the mapping of the positive trend of the area burned annually in Angola, analyzed between 2001 and 2019 (for more details, see Catarino et al., 2020). On the reference map, the significant areas of increasing trends of burned area (at a 95% confidence level) and a 5‐km buffer were classified as 10. On the “highest threat” map, we classified the areas of increasing trends and a buffer of 10 km, while the “lowest threat” map only includes the areas of increasing trends without a buffer area.

#### 2.3.7 Predicted climatic changes: temperature and precipitation

Climate change is expected to increase persistent droughts, heat stress, and insect attacks, associated with an increase in temperature and a decrease in precipitation, which are responsible for substantial tree mortality and damaging effects on forest ecosystems (Anderegg et al., 2015). However, the increase in precipitation can also increase the annual burned area, increasing the vegetation productivity and, consequently, the fuel available for burning (Andela & Werf, 2014).

The predicted changes in annual temperature and precipitation for 2041–2060 were obtained from the Coupled Model Intercomparison Project 6 (CMIP6) downscaling the future climate projections, available at the WorldClim data website (WorldClim, 2020b). We selected the data from Shared Socio‐economic Pathways 3‐70 (SSP 3‐70) because it is in the middle of the range of baseline results produced by energy system models.

The future annual temperature and precipitation were calculated as the mean values of the climate models BCC‐CSM2‐MR, CNRM‐CM6‐1, CNRM‐ESM2‐1, CanESM5, IPSL‐CM6A‐LR, MIROC‐ES2L, MIROC6, and MRI‐ESM2‐0, fully available in the WorldClim (WorldClim, 2020b) for the years 2041–2060. The amplitude of changes was obtained by subtracting the value of current temperatures and precipitation from future values. Finally, we assumed that the pressure induced by climate changes is proportional and increases linearly with the increasing amplitude of changes.

In the temperature layer, the value of each cell ranges from 0 to 10, according to the amplitude of the change, reaching the value of 10 when the temperature changes by 2℃ or more. In this case, the cells’ values were calculated as follows: pressure = |amplitude of change| / 2 × 10. In the “highest threat” map and “lowest threat” map, we assigned the value 10 to amplitudes ≥1.5℃ and ≥2.5℃, respectively.

Similarly, the pressure of precipitation change increases from 0 to 10 up to a maximum of 200 mm; thus, the cells’ values were calculated as follows: pressure = |amplitude of change| / 200 × 10. In the “highest threat” and “lowest threat” maps, the value 10 corresponds to amplitudes ≥150 mm and ≥250mm, respectively.

## 3 RESULTS

### 3.1 Species distribution models

**FIGURE 2:**
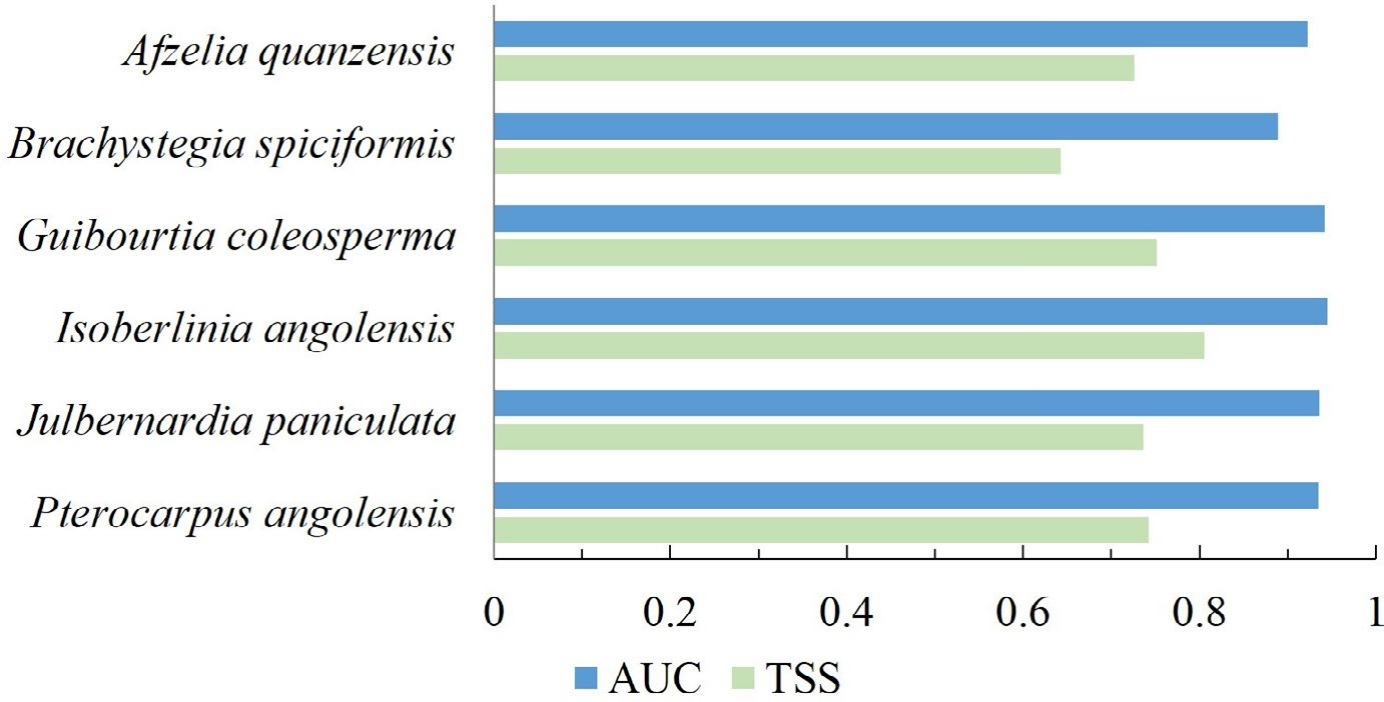
Evaluation of the species ensemble models (mean values) by the area under the curve (AUC) of the receiver operating characteristic and the true skill statistic (TSS)

The evaluation of SDMs was based on TSS and AUC values. The mean AUC values of the individual modeling algorithms ranged from 0.663 to 0.867, and mean TSS values ranged from 0.292 to 0.621. The GBM and RF algorithms had the best evaluations, while the GLM and MaxEnt algorithms presented the poorest performances, in general (Table S1 and Figure S3). The ensemble models were the most accurate for the studied species, as compared with the individual modeling algorithms. Ensemble models showed excellent predictive performances, with mean AUC values between 0.889 and 0.945 and mean TSS values between 0.643 and 0.805 (Figure 2). The importance of environmental predictors varied with the species, but solar radiation in February was one of the most important explanatory variables for five of them (more details in Figure S4).

**FIGURE 3:**
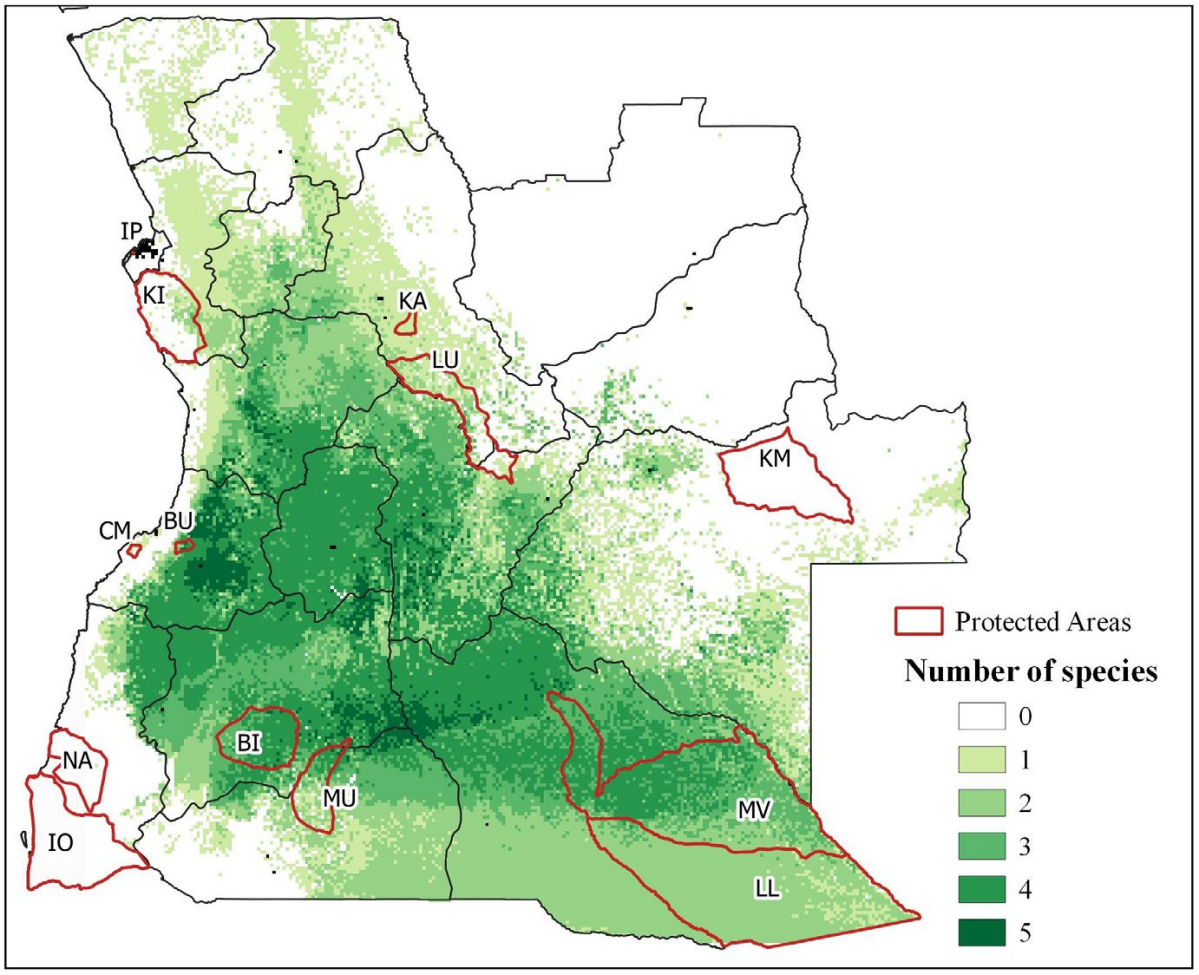
Overlay map of the predicted species distribution, resulting from summing all individual binary maps. Protected areas: BI, Bicuar; BU, Bufalo; KA, Cangandala; CM, Chimalavera; IO, Iona; IP, Ilheu dos Pássaros; KM, Cameia; KI, Quiçama; LL, Luengue‐Luiana; LU, Luando; MV, Mavinga; MU, Mupa; NA, Namibe

Figure 3 shows the overlay map for the predicted species distribution, resulting from the sum of all individual binary maps (presented in Figure S5). The highest number of species is found in central and western provinces, mainly associated with the region of Miombo woodlands. Benguela, Cuanza Sul, Huambo, Huíla, Bié, and Cuando Cubango are the most suitable provinces for the studied species, in general. These provinces have large areas suitable for the simultaneous occurrence of the five species. The northern provinces are the least suitable areas.

### 3.2 Threat index map

**FIGURE 4:**
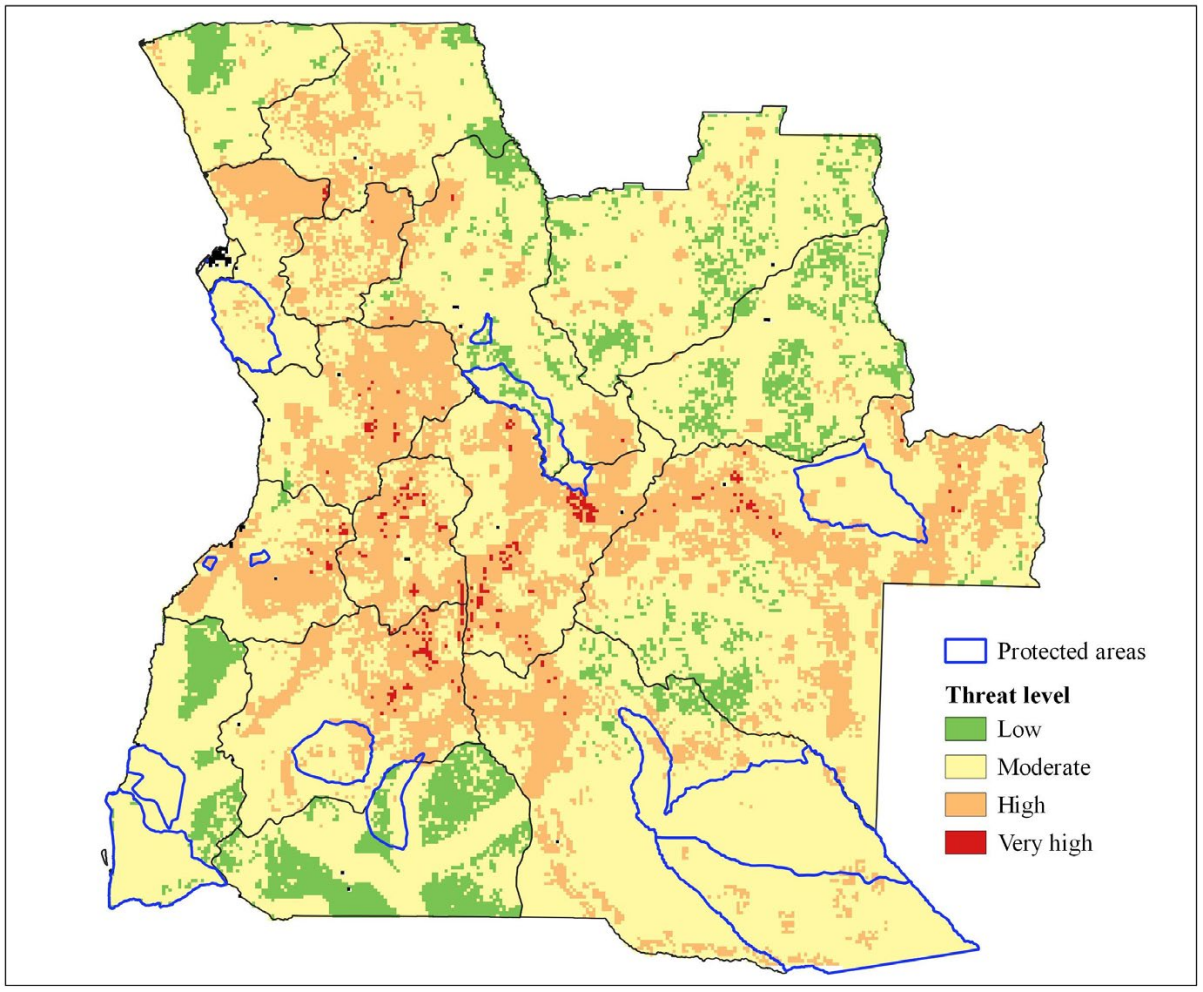
Reference threat index map for Angola timber tree species

The values of the threat index ranged between 7.2 and 73.6 across the country. Based on the index values, the cells were classified into four categories, from “Low” to “Very High.” Figure 4 shows the estimated level of threat in each cell of 25 km^2^. The central regions stand out for the high concentration of cells classified as “High” and “Very high” threat level, and correspond to the provinces of Benguela, Bié, Cuanza Sul, Huambo, Huíla, and Moxico.

In terms of threat level, about 0.5% (ca. 6,820 km^2^) of the country was classified as “Very high,” 23.9% (ca. 295,100 km^2^) as “High,” 66.5% (ca. 827,290 km^2^) as “Moderate,” and 9.1% (ca. 112,360 km^2^) as “Low.” Most of the protected areas (90.6%) correspond to a “Moderate” level of threat, and no cell of "Very High" level was found in these areas.

The sensitivity analysis showed that the threat index map was relatively sensitive to the methodological decisions to create the layers of threat. Comparing the “highest threat” map with the reference map, 13.9% of the grid cells changed from one level of threat to another, 1.5% decreased the level, and 12.3% increased. On the “lowest threat” map, 21.8% of the grid cells changed their level, 1.4% increased, and 19.6% decreased, as compared with the reference map (Figure S6).

### 3.3 Threat level and conservation of Leguminosae timber species

**FIGURE 5:**
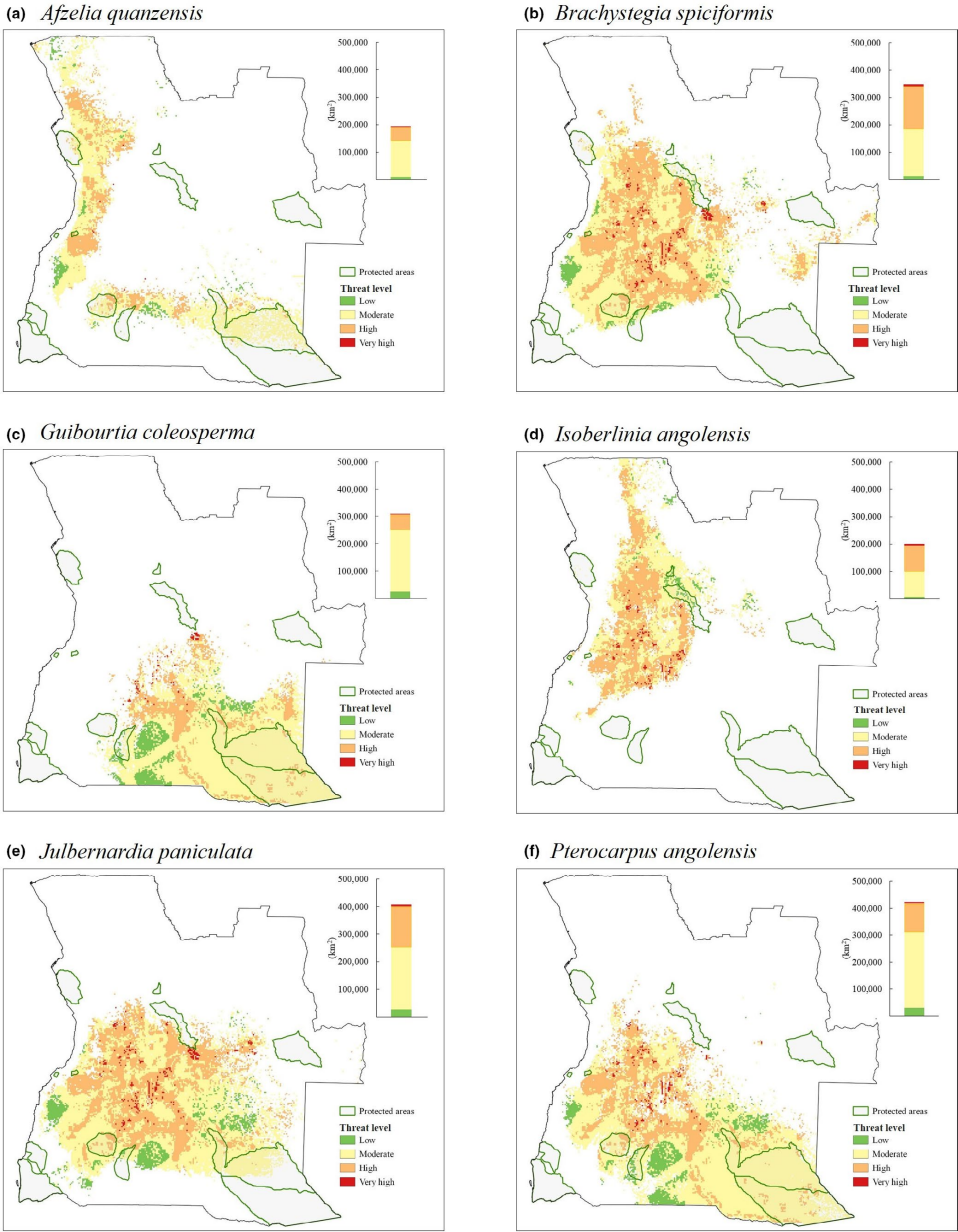
Predicted distribution maps for the six species studied [(a) *Afzelia quanzensis*; (b) *Brachystegia spiciformis*; (c) *Guibourtia coleosperma*; (d) *Isoberlinia angolensis*; (e) *Julbernardia paniculata*; and (f) *Pterocarpus angolensis*] showing the protected areas and the threat level in their suitable area. The color bars on the right of the maps represent the predicted area of distribution (km^2^) under each threat level

The six species studied show different patterns of distribution in Angola, resulting in different levels of threat (Figure 5a‐f, Table S2). *Brachystegia spiciformis* and *I. angolensis* stand out for their largest fraction of suitable area of occurrence in areas of high threat. *Brachystegia spiciformis* (Figure 5b) has almost 47% of its distribution in areas of “High” and “Very high” levels of threat, and only 4.2% of its total area is under protection. *I. angolensis* (Figure 5d) has about 50% of its predicted distribution area under “High” and “Very high” levels of threat, and it is the species with the smallest area under protection, representing 2.9% of its distribution area. A small fraction of the distribution area of *J. paniculata* (Figure 5e) is also under protection (8.2%); however, this species has a very extensive predicted distribution area, covering more than 400,000 km^2^ in Angola. *A. quanzensis* (Figure 5a), *G. coleosperma* (Figure 5c), and *P. angolensis* (Figure 5f) have more than 70% of their predicted area of occurrence under “Moderate” and “Low” threat levels.

Bicuar National Park is the protected area with the highest richness of the studied timber species, including suitable area for the occurrence of *A. quanzensis*, *B. spiciformis*, *J. paniculata,* and *P. angolensis*. Luengue‐Luiana and Mavinga National Parks have great importance for the conservation of *G. coleosperma* and *P. angolensis,* and Luando Integral Nature Reserve and Cangandala National Park are of extreme importance for the protection of *I. angolensis*.

## 4 DISCUSSION

We studied six Leguminosae timber species native to Angola, which are valuable resources for local populations and have been highly exploited for trade in national and/or international markets. All of them also have other uses (i.e., medicinal, forage, and fiber), which increases the pressure on the species. Previous studies (Catarino et al., 2019; Romeiras et al., 2014) had already warned about the increasing exploitation of these trees and the urgent need to take more effective conservation measures.

Concerning the conservation status, all the studied species except for *Guibourtia coleosperma* were evaluated as vulnerable in the list of threatened plants of Angola (Costa et al., 2019). *G. coleosperma* was classified as least concern in Angola. However, the global conservation status does not reflect the current situation in Angola. According to the IUCN Red List (IUCN, 2020), all these species were classified as least concern except for *G. coleosperma*, which was not evaluated. The category least concern may not show the present status of species because the populations of *B. spiciformis* and *P. angolensis* are reported globally decreasing, as a result of habitat loss and overexploitation (Barstow & Timberlake, 2018; Hills, 2019).

Updated data on spatial distribution are essential for the sustainable management and conservation of species. As such data are very scarce for Angolan flora, we predicted the suitable areas for the occurrence of each species by applying species distribution modeling techniques. Based on AUC values, the performance of models developed with a single algorithm was generally “Good” (between 0.7 and 0.9), increasing to “Very good” (>0.9) in the ensemble models. We also used the TSS evaluation method, which confirmed the reliable performance of our models.

Mapping the main threats is also a critical step toward conservation of species and their habitats. The threat index map presented herein represents the first spatial assessment of the main threats to timber species carried out for Angola, which has one of the highest deforestation rates in sub‐Saharan Africa (Hansen et al., 2003). Although this study focused on trees of the Leguminosae family, the threat index map could be generalized to species from other plant families with functional patterns similar to the species analyzed.

We quantified the potential pressure of eight threat factors on the native timber species, including two concerning climatic change and six related to human activity. The anthropogenic factors had a greater weight in the index than climatic changes, and this is in agreement with other recent studies focused on tree species, which suggest that anthropogenic pressures result in greater threats to species populations than climate change (Fremout et al., 2020; Manchego et al., 2017). Human activities have long had a strong impact on Angolan natural resources. Huntley (1974) already described the vegetation and soils around Angolan cities as degraded areas. The lack of fossil fuels led populations to cut down many trees for the production of firewood and charcoal, resulting in large areas of deforested Miombo. Charcoal production has replaced food production as a source of income in some rural zones, increasing the deforested area, especially in areas with high population density in the provinces of Huambo, Bié, and Huíla (PNUD, 2012).

Over the last five years, and without monitoring, the trade of timber products strongly increased, in part due to the effort to find alternative sources of income after the global drop in oil prices (Mendelsohn, 2019). In 2016, the Angolan government increased the concessions for logging in the country, and Chinese companies have massively extracted hardwoods, causing the deforestation of vast areas of Miombo woodlands (Huntley et al., 2019). In 2018, an important conservation measure was taken when Angola banned the cutting of *G. coleosperma* for two years, to relieve pressure caused on them by the extraction of volumes above the quotas legally established in 2016 and 2017 (Diário da República de Angola, Decreto Executivo n. 278/18, 07 August 2018, p. 4108–4109). However, such measures are very rare and the conservation of native timber species is not the main target of forest management.

Although the Angolan government recognizes that the uncontrolled use of wood for commercial purposes led to the loss of key forests in the country, resulting in the loss of species diversity and the impoverishment of local human communities (PNUD, 2012), measures to ensure the sustainable management and forest recovery are still very scarce. As revealed by the threat index map, the region of Huambo and Angola's central plateau is among the areas most exposed to overexploitation and tree cover loss. According to Palacios et al. (2015), this region was originally covered by Miombo woodlands, but between 2002 and 2015 its area decreased from 78% to 48% and converted to cropland. Schneibel et al. (2013) also documented similar patterns in western Cuando Cubango, eastern Huila, and eastern Huambo. The current increasing trends in burned area are also associated with these events (Catarino et al., 2020).

According to Morishima and Akasaka (2010), changes in climate are already observed in southern Africa since the 1980s, with a significant decrease in rainfall from December to March, and an increase in temperature from July to October. Future climate changes could be particularly threatening for some of the species studied; for instance, a significant reduction in *A. quanzensis* and *B. spiciformis* distribution areas is expected until 2050 due to climate change (Jinga & Ashley, 2019).

Considering the limited resources available in Angola, it is essential to identify priority areas for habitat restoration and species conservation. In this study, we identified two main areas of high priority:

1. Central and eastern area of Benguela province, in the municipalities of Caiambambo, Cubal, and Bocoio, where five of the studied species occur. This area is mainly classified as of “High” threat due to a relatively high population density, large areas of cropland, proximity to roads, and overexploitation.
2. The bordering zone between Huíla, Huambo, and Bié provinces, namely the municipalities of Tchicala‐Tcholoanga, Catchiungo, Chitembo, and North Kuvango. This area is highly affected by overexploitation of timber, proximity from the roads, croplands, and predicted temperature changes. The region has large areas of “High” and “Very high” threat level but no protected areas.

Angolan protected areas generally have lower levels of threat, with more than 90% of the area classified as “Moderate” threat. This may indicate that protected areas are being effective in reducing factors that threaten biodiversity. However, our work suggests that their geographical location may not be the most adequate for the protection of timber species, since the richness of these species within protected areas tends to be low. Most Angolan protected areas were established to protect large mammals in colonial times, rather than native vegetation (Romeiras et al., 2014). The extended war (1975–2002) caused negligent management of the areas, endangering the conservation of many species and their habitats (Huntley et al., 2019).

Two main limitations were identified in this study. First, the list of potential threats to timber species is not complete. For instance, invasive plant species, pollution, and nomadic grazing were not included in the threat index map because there is no accurate spatial information available on these subjects. Nevertheless, we believe that these pressures could be strongly associated with others that we included in our study, such as population density and distance from the roads, and consequently, their inclusion would not much affect our final results. Second, the map of threat index measures the level of pressure at which the studied species could be exposed, not the species response to those threats. Thus, further studies on species’ vulnerability to threats could be important to predict their future distribution area, allowing more efficient planning of conservation measures under the effects of climate change.

## CONFLICT OF INTEREST

The authors declare no conflicts of interest.

## AUTHOR CONTRIBUTIONS

**Silvia Catarino:** Conceptualization (equal); Data curation (equal); Formal analysis (equal); Investigation (lead); Methodology (equal); Software (equal); Writing‐original draft (lead); Writing‐review & editing (equal). **Maria M. Romeiras:** Conceptualization (equal); Methodology (supporting); Writing‐review & editing (equal). **José M. C. Pereira:** Conceptualization (equal); Methodology (supporting); Writing‐review & editing (equal). **Rui Figueira:** Conceptualization (equal); Data curation (equal); Formal analysis (equal); Methodology (equal); Software (equal); Writing‐review & editing (equal).

## Supporting information

Supplementary Material

Supplementary Material

## Data Availability

The full dataset of species occurrence records is deposited in Zenodo Repository: https://doi.org/10.5281/zenodo.4752197. The R Package to run Biomod2 is freely available at https://github.com/biomodhub/biomod2.
