# Supplementary material for: Assessing the conservation of Miombo timber species through an integrated index of anthropogenic and climatic threats
Source: Ecol Evol. 2021 Jun 22;11(14):9332–48. doi: 10.1002/ece3.7717 (PMC8293741; doi:10.1002/ece3.7717)
Supplement: Supplementary file 1 — Supplementary Material [file ECE3-11-9332-s002.pdf]

Supplementary Information – Tables and Figures

Additional Tables

Table S1. Evaluation of the individual modelling algorithms (mean values) by the area under the curve (AUC) of the receiver operating characteristic and the true skill statistic (TSS).

Species [a]	AQ		BS		GC		IA		JP		PA	
**Algorithms [b]**	**TSS**	**AUC**	**TSS**	**AUC**	**TSS**	**AUC**	**TSS**	**AUC**	**TSS**	**AUC**	**TSS**	**AUC**
**GLM**	0.372	0.712	0.425	0.753	0.389	0.708	0.398	0.721	0.527	0.807	0.319	0.694
**GBM**	0.438	0.762	0.526	0.809	0.432	0.754	0.450	0.764	0.611	0.864	0.413	0.763
**MARS**	0.379	0.716	0.447	0.769	0.391	0.707	0.420	0.728	0.583	0.840	0.375	0.733
**RF**	0.422	0.764	0.537	0.821	0.440	0.762	0.466	0.776	0.621	0.867	0.453	0.783
**MAXENT**	0.352	0.688	0.418	0.748	0.406	0.717	0.419	0.731	0.498	0.791	0.292	0.663
**ENSEMBLE**	0.726	0.922	0.643	0.889	0.751	0.942	0.805	0.945	0.736	0.936	0.742	0.935

[a] **Species: AQ**, *Afzelia quanzensis*; **BS**, *Brachystegia spiciformis*; **GC**, *Guibourtia coleosperma*; **IA**, *Isoberlinia angolensis*; **JP**, *Julbernardia paniculata*; and **PA**, *Pterocarpus angolensis*.

[b] **Algorithms**: **GLM,** generalized linear models; **GBM,** generalized boosted regression models; **MARS**, multivariate adaptive regression splines; **RF**, random forest; **MAXENT**, MaxEnt; and **ENSEMBLE**, ensemble models.

Table S2. Distribution area with detailed information on the threat level and protected area for each studied species.

**Threat level**	***A. quanzensis*** Distribution area		Distribution inside protected area		***B. spiciformis*** Distribution area		Distribution inside protected area		***G. coleosperma*** Distribution area		Distribution inside protected area	
	**(km^2)**	**(%)**	**(km^2)**	**(% of total area)**	**(km2)**	**(%)**	**(km2)**	**(% of total area)**	**(km2)**	**(%)**	**(km2)**	**(% of total area)**
**Low**	9942	5.19	90	0.05	13623	3.92	1279	0.37	25720	8.32	808	0.26
**Moderate**	131519	68.67	34383	17.95	170817	49.18	10481	3.02	225220	72.87	84051	27.19
**High**	49914	26.06	2963	1.55	156947	45.19	2940	0.85	56602	18.31	5297	1.71
**Very High**	157	0.08	0	0	5948	1.71	0	0	1526	0.49	0	0
**Total**	**191532**	**100**	**37436**	**19.55**	**347335**	**100**	**14700**	**4.23**	**309068**	**100**	**90156**	**29.17**

**Threat level**	***I. angolensis*** Distribution area		Distribution inside protected area		***J. paniculata*** Distribution area		Distribution inside protected area		***P. angolensis*** Distribution area		Distribution inside protected area	
	**(km^2)**	**(%)**	**(km^2)**	**(% of total area)**	**(km^2)**	**(%)**	**(km^2)**	**(% of total area)**	**(km2)**	**(%)**	**(km2)**	**(% of total area)**
**Low**	6598	3.32	1369	0.69	27763	6.82	426	0.1	30972	7.33	1234	0.29
**Moderate**	93589	47.02	4197	2.11	225355	55.36	29266	7.19	279196	66.06	85756	20.29
**High**	95564	48.02	135	0.07	148037	36.37	3479	0.85	108536	25.68	7159	1.69
**Very High**	3277	1.65	0	0	5903	1.45	0	0	3950	0.93	0	0
**Total**	**199028**	**100**	**5701**	**2.86**	**407058**	**100**	**33171**	**8.15**	**422654**	**100**	**94149**	**22.28**

Additional Figures

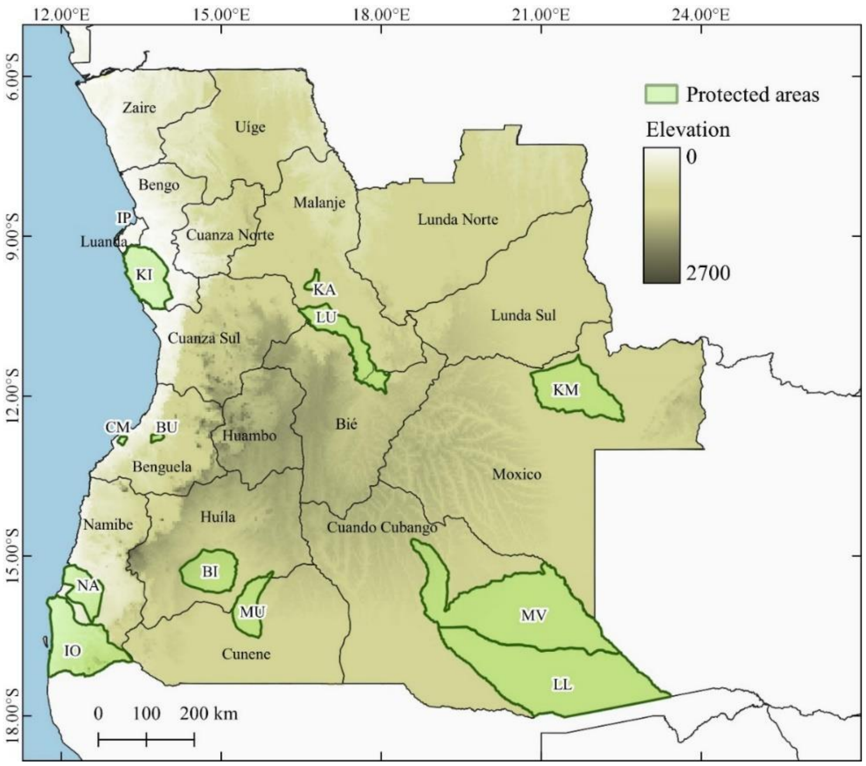

Figure S1. Study area, with details on the provinces of Angola (excluding Cabinda) and national protected areas system (green areas). Protected areas: BI, Bicuar; BU, Bufalo; KA, Cangandala; CM, Chimalavera; IO, Iona; IP, Ilheu dos Pássaros; KM, Cameia; KI, Quiçama; LL, Luengue-Luiana; LU, Luando; MV, Mavinga; MU, Mupa; NA, Namibe.

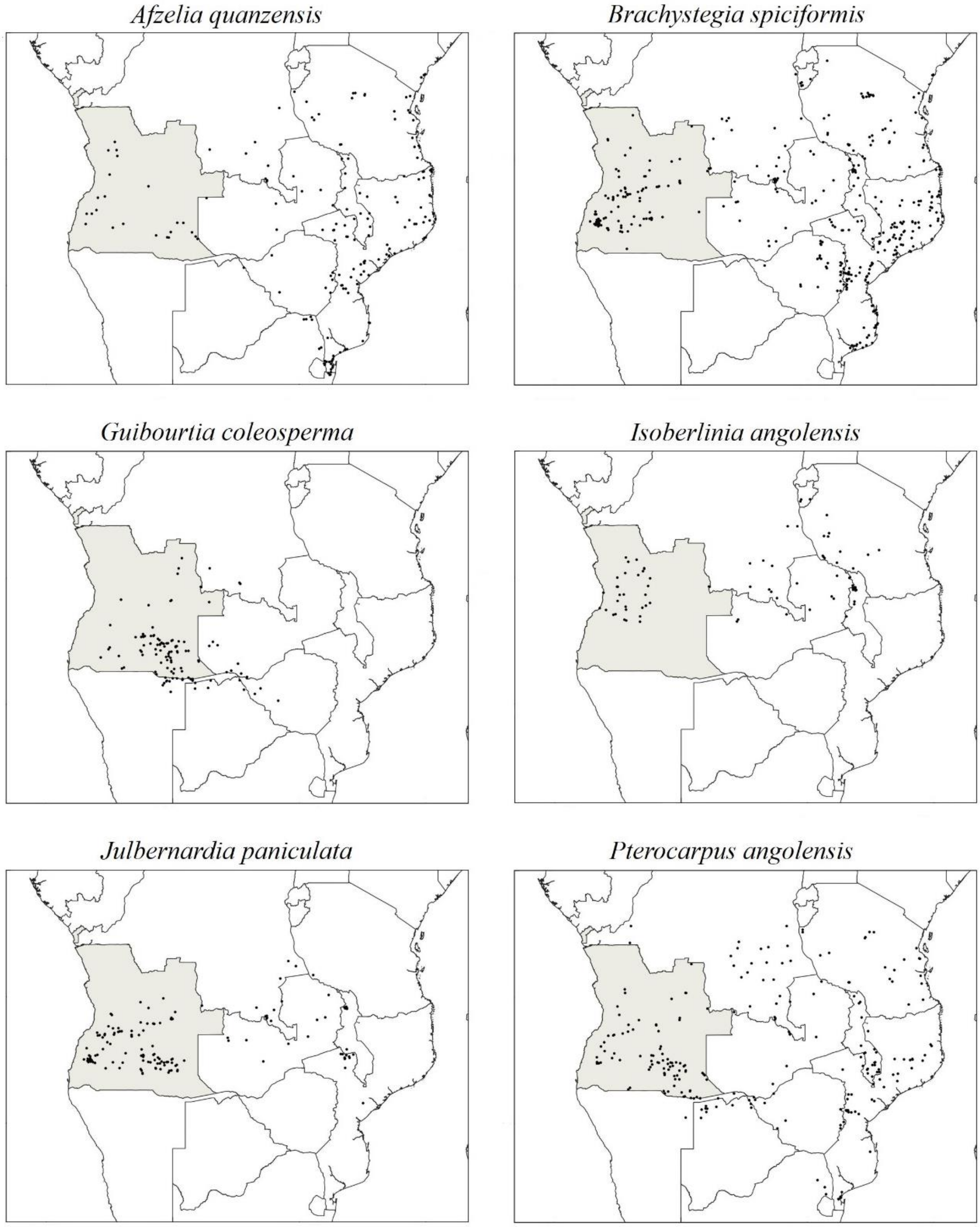

Figure S2. Occurrence records of Leguminosae timber species used to build de species distribution models: *Afzelia quanzensis* (n=172), *Brachystegia spiciformis* (n=370), *Guibourtia coleosperma* (n=112), *Isoberlinia angolensis* (n=79), *Julbernardia paniculata* (n=143), *Pterocarpus angolensis* (n=227).

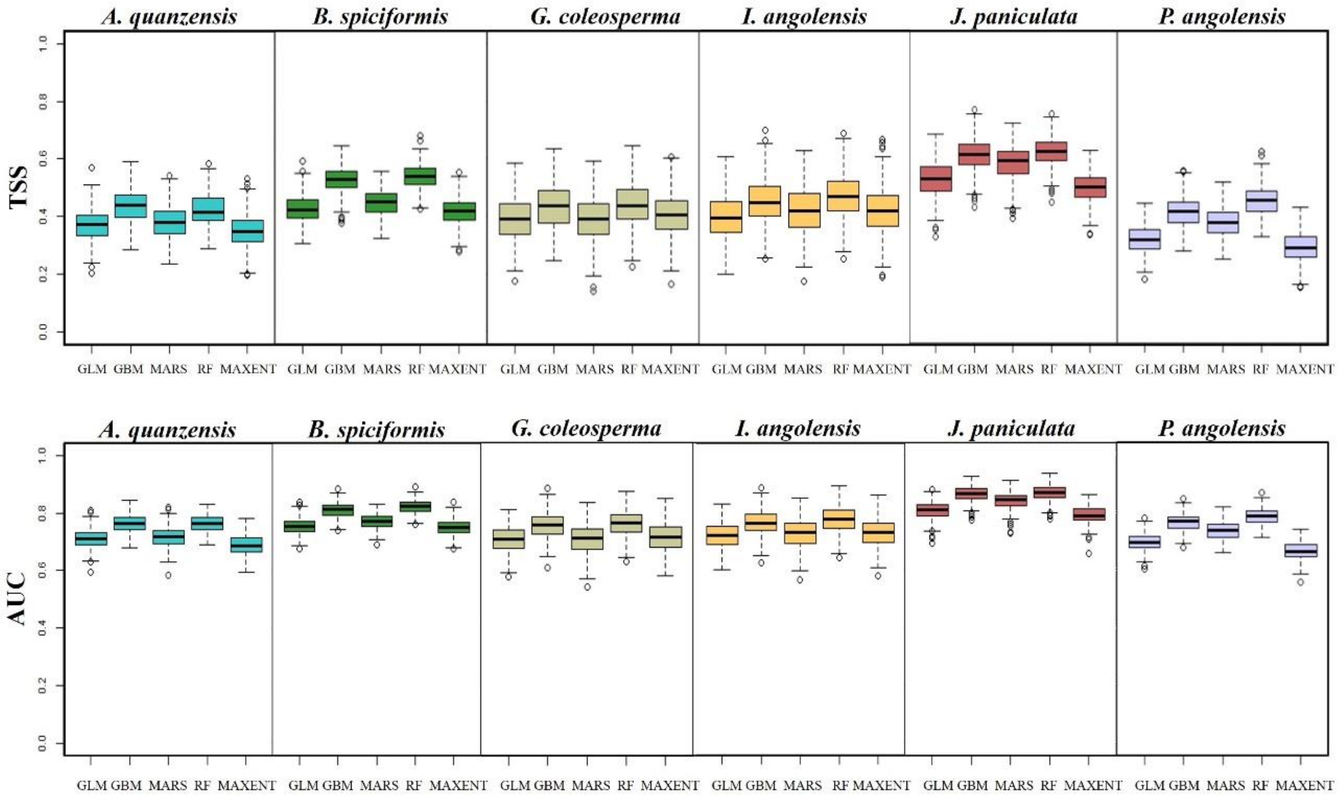

Figure S3. Evaluation of the individual modelling algorithms (mean values) by the area under the curve (AUC) of the receiver operating characteristic and the true skill statistic (TSS). Algorithms: GLM, generalized linear models; GBM, generalized boosted regression models; MARS, multivariate adaptive regression splines; RF, random forest; MAXENT, MaxEnt; and ENSEMBLE, ensemble models.

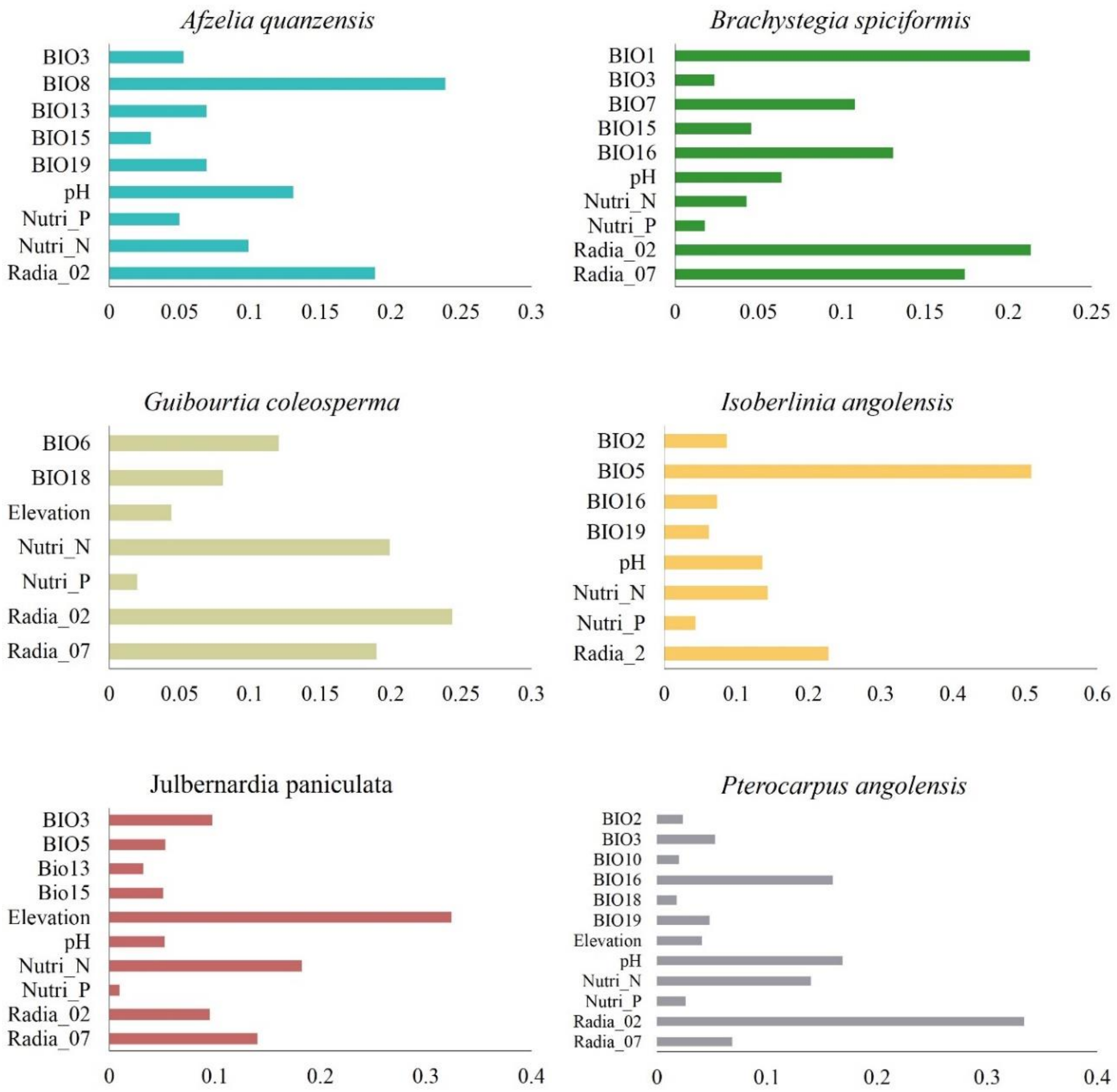

Figure S4. Mean values of relative explanatory variables used to predict the species distribution.

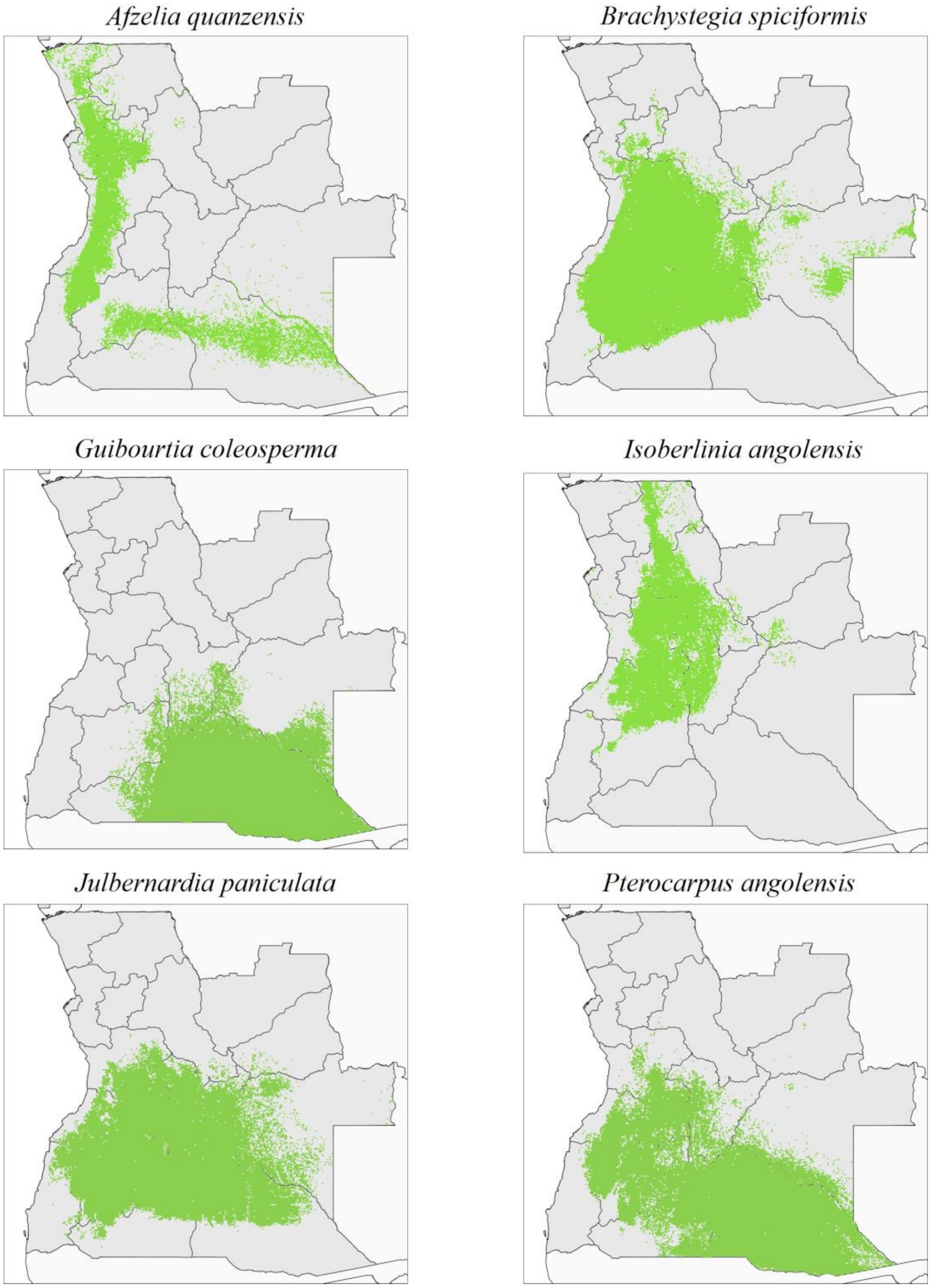

Figure S5. Binary maps of potential distribution of Leguminosae timber species in Angola predicted by ensemble species distribution models.

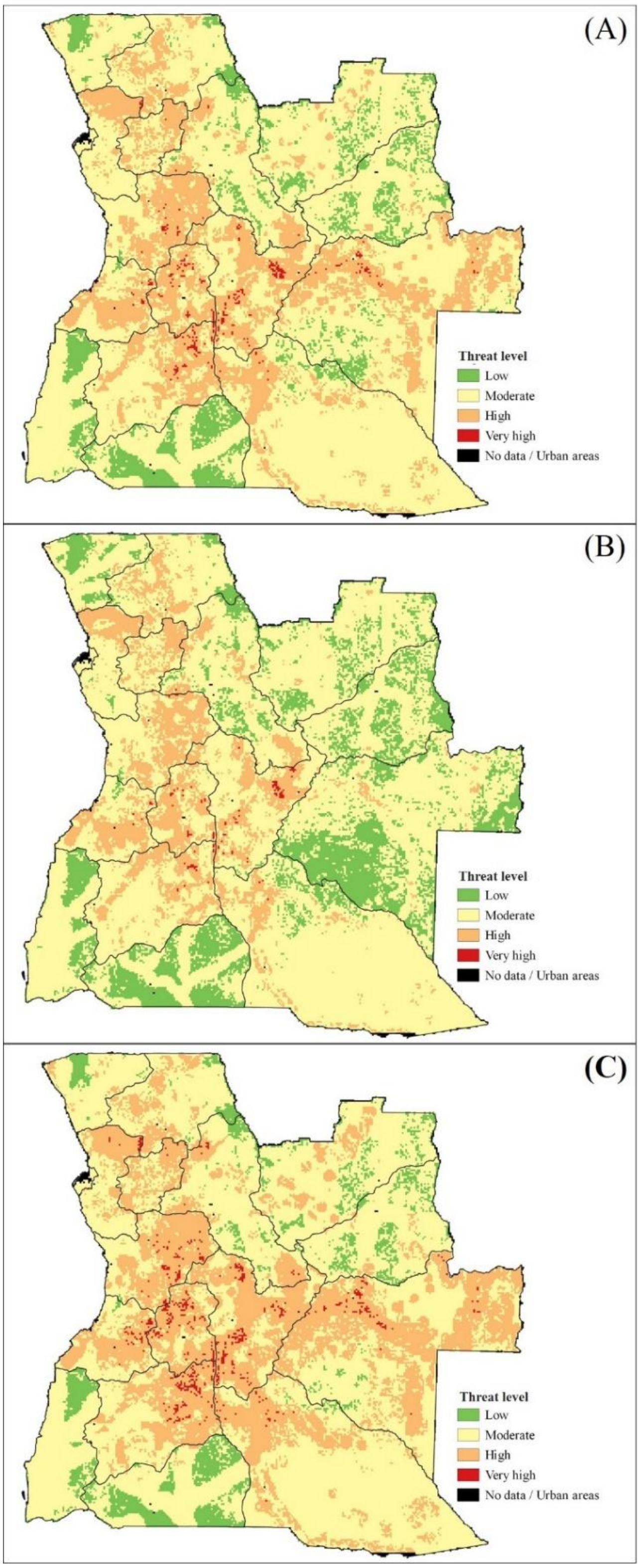

Figure S6. Maps of threat index for timber trees in Angola: (A) Reference map; (B) "Lowest threat" map; and (C) "Highest threat" map.
