# Supplementary material for: Assessing the conservation of Miombo timber species through an integrated index of anthropogenic and climatic threats
Source: Ecol Evol. 2021 Jun 22;11(14):9332–48. doi: 10.1002/ece3.7717 (PMC8293741; doi:10.1002/ece3.7717)
Supplement: Supplementary file 2 — Supplementary Material [file ECE3-11-9332-s001.pdf]

Supplementary Information – ODMAP protocol

Data S1. ODMAP protocol with details of the modelling process, following recommendations of Zurell et al. (2020).

ASSESSING THE CONSERVATION OF MIOMBO TIMBER SPECIES THROUGH AN INTEGRATED INDEX OF ANTHROPOGENIC AND CLIMATIC THREATS

– ODMAP Protocol –

Sílvia Catarino, Maria M. Romeiras, José M. C. Pereira, Rui Figueira

2021-02-20

Overview

Authorship

Contact: scatarino@isa.ulisboa.pt

Study link: N/A

Model objective

Model objective: Mapping and interpolation

Target output: Habitat suitability maps and binary maps of potential presence

Focal Taxon

Focal Taxon: *Afzelia quanzensis, Brachystegia spiciformis, Guibourtia coleosperma, Isoberlinia angolensis, Julbernardia paniculata, and Pterocarpus angolensis*

Location

Location: Sub-Saharan Africa, focused in the Republic of Angola

Scale of Analysis

Spatial extent: 37.6, 63.5, -47.0, -25.4 (xmin, xmax, ymin, ymax)

Spatial resolution: 5 km

Temporal extent: 1970 – 2000

Temporal resolution: N/A

Boundary: natural

Biodiversity data

Observation type: GBIF data

Response data type: point occurrence, presence-only

Predictors

Predictor types: climatic, topographic, edaphic

Hypotheses

Hypotheses: We aim to predict the potential distribution of the studied species to analyse the level of threat within their distribution area.

Assumptions

Model assumptions: We assumed that species are at pseudo-equilibrium with the environment; relevant ecological predictors of species distributions are included; and the presence records represent the suitable habitat of the species. Our methods were designed to address the biased distribution of the species records and the lack of absence data.

Algorithms

Modelling techniques: Generalized linear models (GLM), multivariate adaptive regression splines (MARS), Generalized boosted models (GBM), Random forest (RF) and MaxEnt.

Model complexity: We used an ensemble modelling approach to reduce the uncertainty of predictive single models by combining them and excluding the results with low predictive performance, producing more robust predictions.

Model averaging: The ensemble models combine five different algorithms by the "mean" consensus method. Only single models with AUC ≥ 0.7 were included in ensemble model building.

Workflow

Model workflow: We selected 25 ecological variables as possible predictors to fit the models. To avoid overweighting the analysis with bioclimatic variables, we performed an exploratory modelling exercise to selected the three variables related to temperature and the three variables related to precipitation that most contributed to the distribution model of each species. Then, the Variance Inflation Factor (VIF) was used to reduce multicollinearity between the remaining predictors, using R version 3.6.0. (R Development Core Team, 2020) and "usdm" package (Uncertainty Analysis for SDMs) version 1.1-18 (Naimi et al., 2014). The potential distribution of each species was predicted through an ensemble modelling approach performed with the "biomod2" package (BIOdiversity MODelling- Biomod2) version 3.3-7.1 (Thuiller et al., 2020). We fitted the SDMs using an ensemble of five different modelling algorithms (GLM, GBM, MARS, RF, and MaxEnt), these algorithms were selected based on their superior performance during an exploratory modelling exercise. As our data are presence-only, we generated three different sets of pseudo-absences, where one-third of the available background modelling cells were randomly sampled and used as pseudo-absences. For each

species were produced 1500 individual models. We chose the "mean" consensus method for ensemble model building and only single models with AUC ≥ 0.7 were included.

Software

Software: R (version 3.6.0) with packages "usdm" and "biomod2"

Code availability: R Scripts used for this study can be provided on request.

Data availability: Presence records of the studied species are available in GBIF database and are presented in Figure S2. Environmental predictors can be retrieved from online databases cited in Methods section.

Data

Biodiversity data

Taxon names: *Afzelia quanzensis* Welw., *Brachystegia spiciformis* Benth., *Guibourtia coleosperma* (Benth.) J. Léonard, *Isoberlinia angolensis* (Welw. ex Benth.) Hoyle & Brenan, *Julbernardia paniculata* (Benth.) Troupin, and *Pterocarpus angolensis* DC.

Taxonomic reference system: Followed the Plants of the World Online (POWO, 2020).

Ecological level: species

Data sources: The occurrence records were gathered from GBIF - Global Biodiversity Information Facility:
A. quanzensis (https://doi.org/10.15468/dl.byr43z)
B. spiciformis (https://doi.org/10.15468/dl.sxjqvm)
G. coleosperma (https://doi.org/10.15468/dl.3eg7qu)
I. angolensis (https://doi.org/10.15468/dl.3eg7qu)
J. paniculata (https://doi.org/10.15468/dl.4wtck5)
P. angolensis (https://doi.org/10.15468/dl.eqq3p7)

Sampling design: Specific sampling design and sampling effort unknown.

Sample size: *A. quanzensis* (n=172), *B. spiciformis* (n=370), *G. coleosperma* (n=112), *I. angolensis* (n=79), *J. paniculata* (n=143) and *P. angolensis* (n=227)

Clipping: Republic of Angola, excluding the northern enclave of Cabinda.

Cleaning: The records were analysed in terms of quality and accuracy, duplicate records were deleted and only records with less than 5 km of spatial uncertainty were included. We also applied a 5 km spatial filter to decrease sampling bias, reducing overfitting and increasing the models' performance.

Absence data: N/A

Background data: We generated three different sets of pseudo-absences, where one-third of the available background cells were randomly sampled.

Data partitioning

Training data: We randomly selected 70% of the data for model building.

Validation data: We randomly selected 30% of the data for validation.

Predictor variables

Predictor variables: We selected 25 ecological variables as possible predictors to fit the models, including 19 bioclimatic variables, two solar radiation variables, three variables characterizing soil and elevation.

Data sources: Bioclimatic variables and mean solar radiation were downloaded from WordClim 2.0 (WorldClim, 2020a). Soil data were downloaded from World Soil Information (ISRIC, 2020), and elevation data were downloaded from CGIAR-CSI Consortium for Spatial Information (CGIAR-CSI, 2020).

Spatial extent: 180, 180, 60, 90 (xmin, xmax, ymin, ymax)

Spatial resolution: The resolution of the climate data was 2.5 minutes, the resolution of soil variables was and 250 m and the resolution of altitude data was 90 m.

Coordinate reference system: WGS 84, EPSG: 4326

Temporal extent: Climate data: 1970 – 2000

Data processing: All environmental data layers were resampled to a spatial resolution of 5 km.

Transfer data

Data sources: N/A

Model

Multicollinearity

Multicollinearity: We used the Variance Inflation Factor (VIF) to assess and reduce multicollinearity between the variables (VIF scores < 10).

Model settings

The models were processed with the default settings for each modelling technique and the following options: equal weight of background absences and occurrences; 10000 maximum interactions; 100 replicate runs for each set of "pseudo-absences" and for each model technique; the occurrence data were randomly split into 70% training data and 30% test data to evaluate the predictive performance of the models; and 10 permutations to estimate variable importance.

Model settings (extrapolation): N/A

Model estimates

Coefficients: N/A

Variable importance: The importance of variables was calculated using "variables_importance" function of "biomod2" package. This function shuffles a single variable of the given data set and computes a correlation of the model predictions obtained with the initial dataset and with the shuffled data set.

Model selection - model averaging - ensembles

Model ensembles: We chose the "mean" consensus method, as it is reported to provide significantly more robust predictions than the other consensus methods (Marmion et al., 2009). Only single models with AUC ≥ 0.7 were included in ensemble model building.

Analysis and Correction of non-independence

Spatial autocorrelation: N/A

Threshold selection

Threshold selection: To convert the continuous probability maps into binary maps we applied the 10th percentile threshold.

Assessment

Performance statistics

Performance on validation data: Predictive model performance on validation data was assessed area under the receiver operating characteristic curve (AUC) and true skill statistic (TSS).

Plausibility check

Response shapes: No plausibility checks conducted.

Expert judgement: Predicted maps of the species distribution areas were checked by experts.

Prediction

Prediction output

Prediction unit: Our models are not used to make spatial or temporal predictions to new sites.

Uncertainty quantification

N/A
